# New Microsatellite Markers for Examining Genetic Variation in Peripheral and Core Populations of the Coastal Giant Salamander (*Dicamptodon tenebrosus*)

**DOI:** 10.1371/journal.pone.0014333

**Published:** 2010-12-15

**Authors:** Rachael Y. Dudaniec, Andrew Storfer, Stephen F. Spear, John S. Richardson

**Affiliations:** 1 Department of Forest Sciences, University of British Columbia, Vancouver, Canada; 2 School of Biological Sciences, Washington State University, Pullman, Washington, United States of America; University of Sydney, Australia

## Abstract

The Coastal Giant Salamander (*Dicamptodon tenebrosus*) is classified as threatened at the northern periphery of its range in British Columbia (BC), Canada, primarily due to forestry practices and habitat fragmentation. Characterising dispersal behaviour and population connectivity is therefore a priority for this region, while genetic differentiation in core versus peripheral locations remains unstudied in this wide-ranging species. We present seven new polymorphic microsatellite markers for use in population genetic analyses of *D. tenebrosus*. We examine locus characteristics and genetic variation in 12 streams at the species' northern range limit in BC, and within two regions representing sub-peripheral (North Cascades) and core localities (South Cascades) in Washington State, United States. In BC, the number of alleles per locus ranged from 2–5 and observed heterozygosity ranged from 0.044–0.825. Genetic differentiation was highest between BC and the South Cascades, and intermediate between BC and the North Cascades. Across loci, mean allelic richness was similar across regions, while private allelic richness was highest in the core locality (corrected for sample size). These new microsatellite loci will be a valuable addition to existing markers for detailed landscape and population genetic analyses of *D. tenebrosus* across its range.

## Introduction

Species at the periphery of their range may show reduced genetic diversity that can limit microsatellite variation and the potential for detailed genetic analyses that are relevant for conservation [1]. The stream-breeding Coastal Giant Salamander (*Dicamptodon tenebrosus*) is endemic to the Pacific North-west coast of North America, from northern California up to an approximately 100 km^2^ area in the Chilliwack Valley of southern British Columbia (BC), Canada. In Canada, *D. tenebrosus* is designated as *Threatened* and is on the provincial *Red List* in BC (COSEWIC 2000: http://www.sararegistry.gc.ca/species/speciesDetails), due to high susceptibility to decline from habitat degradation owing to forestry practices [1], [2]. Such peripheral populations are uniquely positioned to aid conservation management throughout a species' range, as they can provide information regarding adaptive potential and local adaptation at environmental margins [1]. We describe seven new polymorphic loci specific for *D. tenebrosus*, complementing existing microsatellite loci developed for *D. tenebrosus* [2], [3] and those previously cross-amplified from *D. copei* [4]. We examine locus characteristics in populations of *D. tenebrosus* at their northern range limit to evaluate genetic variation at these new microsatellite markers. We also provide locus characteristics for two regions in Washington State (WA) to examine for differences in allelic diversity, heterozygosity, and genetic differentiation between peripheral populations in BC, with sub-peripheral (North Cascades) and core (South Cascades) regions of the species' range. These new loci have potential use in other *Dicamptodon* species and combined with existing loci, will enable range-wide genetic analyses using high-resolution microsatellite data.

## Materials and Methods

### Ethics statement

This research was conducted with approval of the Animal Care Committee of the University of British Columbia (permit A08-0241) in accordance with the Canadian Council on Animal Care.

Samples were collected from *D. tenebrosus* throughout the Chilliwack Valley of British Columbia in Canada (∼70 km^2^ in area), and from two regions in Washington State, United States (each ∼30 km^2^ in area). All individuals were anaesthetised in a 0.05% solution of MS-222 (0.5 g/L), before a 2–4 mm^2^ sample of tail tissue was cut from the tail tip and preserved in 90% ethanol. Individuals were immediately recovered in a stream water bath for 10–20 minutes before being returned to their capture location. Genomic DNA was extracted from British Columbian samples using a standard phenol-chloroform extraction method [5] and from WA samples using a QIAGEN DNeasy 96 extraction kit. An enriched library was made by Ecogenics GmbH (Zurich, Switzerland) from size-selected genomic DNA ligated into SNX forward/SNX reverse linker [6] and enriched by magnetic bead selection with biotin-labelled (CT)_13_, (GT)_13_, (GTAT)_7_ and (GATA)_7_ oligonucleotide repeats [7], [8]. Of 528 recombinant colonies screened, 330 gave a positive signal after hybridization. Plasmids from 100 positive clones were sequenced and primers were designed for 33 microsatellite inserts, of which 25 were tested for polymorphism. Polymorphism at seven loci (Table 1) was established by preliminary testing undertaken by Ecogenics GmbH (Zurich, Switzerland) using 42 randomly selected individuals. We present locus characteristics and examine genetic diversity in a larger sample size from 12 streams (i.e. populations) sampled within the northern range limit of *D. tenebrosus* in British Columbia (Latitude: 49°4′10; Longitude: -121°53′00). We then compare BC locus characteristics with those from individuals collected within the North Cascades (NC) (Latitude: 48°42′00; Longitude: -121°12′00), and the South Cascades (SC) (Latitude: 45°41′00; Longitude: -122°08′00) of Washington State, which are located approximately 60 km and 350 km south of the Chilliwack Valley respectively. NC may therefore be regarded as sub-peripheral, and SC as core within the entire range of *D. tenebrosus*. Individuals with missing genetic data were excluded from the dataset. Genotypes from each region (BC, NC and SC) were screened for genetic relatedness in the program COLONY 2.0 [9] and full sibs were removed from each population to minimise the effect of relatedness on allele frequencies. This resulted in a total sample size of 291 individuals (16–32 per stream, for 12 streams) in BC, 22 for NC (4 streams) and nine for SC (two streams). Streams are analysed separately for BC, and are pooled by region in Washington State (NC and SC) due to small sample sizes per stream and a less extensive sampling area. Furthermore, strong evidence for high dispersal and minimal population genetic structuring of *D. tenebrosus* in the Cascade Mountains [10] suggests the sampling areas in WA represent panmictic populations.

**Table 1:** Locus name, clone name, GenBank accession number, primer sequence with fluorescent dye label forward (F) and reverse (R), clone repeat unit and primer annealing temperature (T_a_°C) for *D. tenebrosus* microsatellite loci.

Locus	Clone name	Genbank accession number	Primer sequence (5′-3′)	Clone repeat unit	T_a_°C
Dicten02	020048	GU187896	F:(NED)ACCTGTGGACAGGAGGTTTG R:CATTCCCCTGTGTGGCTAAC	(GT)_12_	57
Dicten11	020320.2	GU187905	F:(FAM)CTCGACCACTTCGGTCAAAC R:TGCCTCTGGATACCTTGTGG	(CATA)_6_	57
Dicten18	020361	GU187909	F:(VIC)CGAATGGAGCACATACAGACC R:TTTCACAGGCTTTTGCAGTG	(ATAC)_12_	57
Dicten20	020363	GU187910	F:(NED)TCGCACTTTATAAATCCCAACAC R:ACCCAACCCCATAAGGTCTC	(TACA)_18_	57
Dicten25	020330	GU187907	F:(VIC)GCTTCTGCGGTGAAGGATG R:AGACCATCCGTAGCATGACC	(ATAC)_14_	57
Dicten27	020355	GU187908	F:(NED)AGGTTCGCGCTATATAAATCC R:GCACCACATGATGTTTGACAG	(ATAC)_18_	57
Dicten29	020326	GU187906	F:(VIC)TTCGTGATATATAACAACCAGCACR:CAAATCAGGCAAATACTTAATGG	(CATA)_5_ (CATG) (CATA)_14_	57

Microsatellite loci were amplified for all individuals by polymerase chain reaction (PCR) using a QIAGEN Multiplex PCR Kit. All loci were labelled with fluorescent dye (forward primer) and multiplexed in a 20 µl reaction on a PTC-100 Thermal Cycler (MJ Research). PCR reagents were: 2x QIAGEN Multiplex PCR Master Mix (1x final concentration), 0.2 µM primer mix (forward and reverse), 5x Q-Solution (0.5x final concentration) and 10-20 ng DNA. Amplification conditions followed the QIAGEN protocol for Multiplex PCR using Q-Solution: 15 min at 95°C (initial activation step), followed by 30 cycles consisting of 94°C for 30 s, 57°C annealing for 1.5 min, 72°C for 1.5 min, and a final extension step at 72°C for 10 min. Electrophoresis of multiplexed PCR products was performed on an ABI3730 automated sequencer (Applied Biosystems) with a LIZ500 size standard run with each sample. Genotypes were manually scored using Genemapper v.3.7 (Applied Biosystems). The presence and frequency of null alleles (Oosterhout method) was examined using MICROCHECKER [11]. Tests for departure from Hardy–Weinberg (HW) equilibrium and linkage disequilibrium were performed using GENEPOP 3.4 [12] and corrected using the sequential Bonferroni procedure [13]. Observed and expected heterozygosity, and the frequency of private alleles in each region were calculated using GenAlex 6.2 [14]. The observed number of alleles in a sample is highly dependent on sample size, therefore allelic richness and private allelic richness was calculated using HP-Rare 1.1 [15] correcting for sample size (n) in each region using the rarefaction method, which fixes n as the smallest number of individuals typed for a locus in a sample [16]. Pairwise Fst was calculated in Microsatellite Analyser 4.2 [17] between all 12 BC populations, NC and SC, and between BC populations pooled with NC and SC, with 10 000 permutations on alleles using only loci in linkage and HW equilibrium. Significant genetic differentiation (Fst) between populations was assessed after Bonferroni correction using a threshold of P<0.05, and significant genotypic differentiation within populations was assessed using GENEPOP 3.4, which tests whether genotypes were drawn from the same distribution in all populations [12].

## Results

None of the loci were in linkage disequilibrium within BC populations, NC or SC after correction for multiple comparisons. There was no evidence for scoring error due to stuttering or large allele dropout in any of the loci within any region. In BC, Dicten27 and Dicten11 deviated from HW equilibrium in 100% and 91.7% of the populations sampled respectively, while all other loci were in HW equilibrium across all BC populations except for Dicten29 in one population (Table S1, Table 2). Dicten27 and Dicten11 will therefore be of limited use in further studies of population substructuring. All loci in NC and SC were in keeping with HW expectations (Table 2). Evidence for null alleles was only found for Dicten27 in BC, as suggested by the excess of homozygotes for most allele size classes. Although similar among regions, observed heterozygosity (*He*) across loci increased with sample size of each region, with mean *He* (± s.e.) being highest in BC (0.321±0.11, n = 291), intermediate in SC (0.302±0.09, n = 22) and lowest in NC (0.266±0.09) (Table 2).

**Table 2:** Summary of locus characteristics for *D. tenebrosus* by region (British Columbia, 12 populations pooled), North Cascades and South Cascades.

Region	*A*	Allele size range	*He*	*Ho*	# Private alleles (frequency range)	Null	HW test
**British Columbia (n = 291)**							*# of streams not in HW (%)*
Dicten02	5	168–190	0.374	0.450	4 (0.002–0.151)	−0.241	0
Dicten11	4	139–149	0.504	0.825	2 (0.009–0.009)	−0.444	11(91.7)
Dicten18	3	118–126	0.044	0.038	0	0.038	0
Dicten20	4	211–225	0.130	0.137	1 (0.002)	−0.071	0
Dicten25	2	193–197	0.063	0.058	0	0.027	0
Dicten27	4	121–141	0.592	0.343	1 (0.138)	0.227†	12 (100)
Dicten29	5	158–174	0.332	0.395	1 (0.010)	−0.157	1 (8.3)
**North Cascades (n = 22)**							*P-value for HW test*
Dicten02	2	178–188	0.416	0.59	1 (0.295)	−0.360	0.121
Dicten11	2	139–147	0.268	0.32	0	−0.216	1.0
Dicten18	2	118–126	0.087	0.09	0	−0.080	1.0
Dicten20	3	203–231	0.606	0.50	0	0.138	0.101
Dicten25	1	193	0.000	0.00	0	0	-
Dicten27	3	133–141	0.492	0.36	0	0.175	0.07
Dicten29	2	158–182	0.087	0.00	0	0.238	0.024*
**South Cascades (n = 9)**							
Dicten02	2	180–188	0.444	0.444	1 (0.667)	0	1.0
Dicten11	2	139–147	0.222	0.198	0	−0.118	1.0
Dicten18	2	122–126	0.111	0.105	0	−0.057	0.860
Dicten20	5	203–231	0.667	0.710	2 (0.056–0.111)	0.053	0.403
Dicten25	2	193–197	0.222	0.198	0	−0.118	1
Dicten27	1	133	0.000	0.000	0	0	-
Dicten29	5	158–182	0.444	0.457	1 (0.056)	0.044	0.548

n =  sample size of individuals, *A* =  number of alleles, *He*  =  expected heterozygosity, *Ho*  =  observed heterozygosity, Null  =  estimated null allele frequency (Oosterhout method), and the number of private alleles per locus and their frequency range for each region. Results of Hardy-Weinberg (HW) equilibrium tests are presented for British Columbia as the number of populations with significant deviation from HW expectations (P<0.05 after Bonferroni correction). Within the North Cascades and the South Cascades, P-values of HW tests are presented for each locus.

* =  significant deviation from HW equilibrium after Bonferroni correction.

† =  evidence for null alleles at this locus.

See Table S1 for population-level locus characteristics in British Columbia.

The mean number of alleles across loci (± s.e.) showed an expected increase with sample size in each region (BC = 3.86±0.40; SC = 2.71±0.61; NC = 2.14±0.26). However, when corrected for sample size (n = 9 samples with 18 genes based on NC), allelic richness in BC was more similar to NC and SC (BC = 2.31±0.31; NC = 2.71±0.61; SC = 1.99±0.25), indicating that estimates of allelic richness in NC and SC may be underestimated due to sample size. Despite a higher number of private alleles in BC (as expected for a greater sample size), their mean frequency was very low (mean 0.04±0.02) compared to NC (0.295±0.0) and SC (0.22±0.15) (Table 2). Furthermore, when corrected for sample size, private allelic richness was highest in SC (0.92) compared to NC (0.25) and BC (0.53).

Pairwise Fst was calculated excluding Dicten 27 and Dicten 11 in all regions. Genetic differentiation (Fst) was significant (P<0.05) for all pairwise regional comparisons, and followed expectations of isolation by distance, with the lowest differentiation between the northern peripheral (BC) and sub-peripheral (NC) regions (Fst = 0.176) The highest genetic differentiation was between the most spatially distant BC and SC regions (Fst = 0.330) and was intermediate between NC and SC (Fst = 0.192). Genetic differentiaton within BC populations was low to moderate and significant for ∼31% of all pairwise comparisons, while all 12 populations differed significantly from NC and SC (Table 3), further emphasising the genetic distinctiveness of the three regions. There was highly significant genotypic differentiation (Fisher's exact test  = P<0.002, df = 14) within populations for all loci except Dicten18 and Dicten27 (Fisher's exact test  = P>0.1, df = 14).

**Table 3:** Pairwise Fst between 12 populations in British Columbia and two regions of Washington State: North Cascades (NC) and South Cascades (SC) calculated for five loci in linkage and Hardy-Weinberg equilibrium, with alleles permuted 10 000 times.

	1	2	3	4	5	6	7	8	9	10	11	12	NC
1													
2	0.089												
3	**0.113**	−0.007											
4	0.065	0.020	0.025										
5	0.066	−0.006	−0.004	0.014									
6	**0.202**	**0.149**	**0.118**	**0.175**	**0.114**								
7	0.066	0.032	0.047	0.002	0.022	**0.249**							
8	0.013	0.079	**0.104**	0.038	0.059	**0.219**	0.028						
9	**0.166**	0.029	0.017	**0.087**	0.035	0.080	**0.132**	**0.167**					
10	**0.157**	0.047	0.045	**0.058**	**0.052**	**0.158**	**0.092**	**0.124**	0.068				
11	0.091	0.030	0.025	0.066	0.020	0.042	**0.103**	**0.110**	0.010	**0.062**			
12	**0.193**	0.027	0.011	**0.090**	0.039	0.123	**0.132**	**0.184**	−0.006	**0.071**	0.043		
NC	**0.260**	**0.172**	**0.169**	**0.167**	**0.176**	**0.256**	**0.214**	**0.221**	**0.157**	**0.076**	**0.160**	**0.174**	
SC	**0.395**	**0.286**	**0.283**	**0.230**	**0.278**	**0.479**	**0.268**	**0.316**	**0.322**	**0.168**	**0.290**	**0.356**	**0.192**

Significant values are in bold after strict Bonferroni correction (P<0.05).

## Discussion

We present seven new polymorphic microsatellite loci for *D. tenebrosus*, and show variation in allelic richness and increasing genetic differentiation between peripheral (BC), sub-peripheral (NC) and core (SC) regions of the species' range. Although our sample sizes restrict conclusions regarding differences in genetic variation between regions, our data indicate that allelic richness is comparable between regions, with rare alleles being more common in the range core. Previous studies at the species' northern periphery have found monomorphism or low genetic diversity in loci for *D. tenebrosus* (range of *He* for three loci  = 0.0-0.24) [2], [3], compared to studies conducted in the South Cascades (range of *He* for nine loci  = 0.18-0.85) [10], yet these studies were not concurrent and used different microsatellite markers. Clearly, a larger sample size of core populations and more genetic markers will be necessary to adequately test the central-peripheral hypothesis in this species [18]. The new markers we present will provide greater power to conduct these analyses, and ensure that genetic structure can be well-characterised across the range of *D. tenebrosus*. However, our data provide the first indication of high genetic differentiation between British Columbian and Washington State regions, lending it to broader questions relating to ecological adaptation in this species. Not only will the new loci provide increased potential for high data resolution in genetic studies of this species, but may also be useful in studies of three other *Dicamptodon* species occurring in the Pacific Northwest (e.g. *D. copei, D. atterimus, D. ensatus*). We suggest these new loci will complement existing loci [2], [4] for conducting analyses of population genetic structure, and the effects of habitat degradation on core and threatened peripheral populations of this stream amphibian.

## Supporting Information

Table S1 Locus characteristics by population, British Columbia
(0.15 MB DOC)
