# Supplementary material for: New Microsatellite Markers for Examining Genetic Variation in Peripheral and Core Populations of the Coastal Giant Salamander (Dicamptodon tenebrosus)
Source: PLoS One. 2010 Dec 15;5(12):e14333. doi: 10.1371/journal.pone.0014333 (PMC3002275; doi:10.1371/journal.pone.0014333)
Supplement: Table S1 — Locus characteristics by population, British Columbia (0.15 MB DOC) [file pone.0014333.s001.doc]

Supporting Information

Table S1. Locus name, number of alleles (*A*), observed (*Ho*) and expected (*He*) heterozygosity, and estimate of null allele frequency (Oosterhout) for *D. tenebrosus* within each stream (n =12) sampled in British Columbia. * = indicates locus was not in Hardy-Weinberg equilibrium after Bonferroni correction; n = sample size per stream.

| **Stream** | **Locus** | **Na** | ***Ho*** | ***He*** | **Null** |
| --- | --- | --- | --- | --- | --- |
| **1** | Dicten02 | 2 | 0.069 | 0.067 | -0.035 |
| n = 29 | Dicten11 | 2 | 0.724 | 0.462 | -0.475 |
|  | Dicten18 | 2 | 0.034 | 0.034 | -0.174 |
|  | Dicten20 | 3 | 0.069 | 0.067 | -0.035 |
|  | Dicten25 | 1 | 0.000 | 0.000 | 0 |
|  | Dicten27* | 4 | 0.345 | 0.530 | 0.208* |
|  | Dicten29 | 2 | 0.655 | 0.441 | -0.196 |
| **2** | Dicten02 | 3 | 0.556 | 0.434 | -0.030 |
| n = 18 | Dicten11* | 3 | 0.833 | 0.545 | -0.384 |
|  | Dicten18 | 1 | 0.000 | 0.000 | 0 |
|  | Dicten20 | 2 | 0.056 | 0.054 | -0.028 |
|  | Dicten25 | 1 | 0.000 | 0.000 | 0 |
|  | Dicten27* | 3 | 0.389 | 0.619 | 0.196* |
|  | Dicten29 | 3 | 0.333 | 0.285 | -0.178 |
| **3** | Dicten02 | 4 | 0.633 | 0.475 | -0.355 |
| n = 30 | Dicten11 | 3 | 0.833 | 0.505 | -0.426 |
|  | Dicten18 | 2 | 0.067 | 0.064 | -0.034 |
|  | Dicten20 | 2 | 0.067 | 0.064 | -0.034 |
|  | Dicten25 | 2 | 0.100 | 0.095 | -0.051 |
|  | Dicten27* | 4 | 0.200 | 0.405 | 0.254* |
|  | Dicten29 | 4 | 0.267 | 0.238 | -0.139 |
| **4** | Dicten02 | 4 | 0.654 | 0.493 | -0.361 |
| n = 26 | Dicten11* | 2 | 0.962 | 0.499 | -0.804 |
|  | Dicten18 | 2 | 0.077 | 0.142 | 0.137 |
|  | Dicten20 | 2 | 0.154 | 0.142 | -0.08 |
|  | Dicten25 | 1 | 0.000 | 0.000 | 0 |
|  | Dicten27* | 4 | 0.346 | 0.600 | 0.218* |
|  | Dicten29 | 3 | 0.615 | 0.501 | -0.177 |
| **5** | Dicten02 | 4 | 0.548 | 0.432 | -0.299 |
| n = 31 | Dicten11 | 2 | 0.871 | 0.492 | -0.641 |
|  | Dicten18 | 2 | 0.032 | 0.032 | -0.016 |
|  | Dicten20 | 2 | 0.065 | 0.062 | -0.033 |
|  | Dicten25 | 2 | 0.097 | 0.148 | 0.113 |
|  | Dicten27* | 4 | 0.484 | 0.678 | 0.159* |
|  | Dicten29 | 2 | 0.387 | 0.312 | -0.217 |
| **6** | Dicten02 | 1 | 0.000 | 0.000 | 0 |
| n =17 | Dicten11* | 2 | 0.941 | 0.498 | -0.758 |
|  | Dicten18 | 1 | 0.000 | 0.000 | 0 |
|  | Dicten20 | 1 | 0.000 | 0.000 | 0 |
|  | Dicten25 | 2 | 0.059 | 0.057 | -0.030 |
|  | Dicten27* | 4 | 0.412 | 0.621 | 0.194* |
|  | Dicten29 | 1 | 0.000 | 0.000 | 0 |
| **7** | Dicten02 | 4 | 0.619 | 0.454 | -0.358 |
| n = 21 | Dicten11* | 2 | 0.905 | 0.495 | -0.691 |
|  | Dicten18 | 1 | 0.000 | 0.000 | 0 |
|  | Dicten20 | 3 | 0.095 | 0.092 | -0.048 |
|  | Dicten25 | 1 | 0.000 | 0.000 | 0 |
|  | Dicten27* | 4 | 0.286 | 0.558 | 0.259* |
|  | Dicten29 | 3 | 0.762 | 0.503 | -0.472 |
| **8** | Dicten02 | 3 | 0.250 | 0.225 | -0.130 |
| n = 32 | Dicten11* | 2 | 0.938 | 0.498 | -0.750 |
|  | Dicten18 | 1 | 0.000 | 0.000 | 0 |
|  | Dicten20 | 3 | 0.219 | 0.198 | -0.114 |
|  | Dicten25 | 2 | 0.031 | 0.031 | -0.016 |
|  | Dicten27* | 4 | 0.313 | 0.617 | 0.257* |
|  | Dicten29 | 2 | 0.813 | 0.482 | -0.567 |
| **9** | Dicten02 | 3 | 0.375 | 0.314 | -0.203 |
| n = 16 | Dicten11 | 3 | 0.875 | 0.555 | -0.428 |
|  | Dicten18 | 2 | 0.063 | 0.061 | -0.032 |
|  | Dicten20 | 2 | 0.125 | 0.117 | -0.065 |
|  | Dicten25 | 2 | 0.063 | 0.061 | -0.032 |
|  | Dicten27* | 4 | 0.188 | 0.373 | 0.239* |
|  | Dicten29 | 1 | 0.000 | 0.000 | 0 |
| **10** | Dicten02 | 4 | 0.800 | 0.583 | -0.434 |
| n = 25 | Dicten11* | 2 | 0.760 | 0.471 | -0.510 |
|  | Dicten18 | 1 | 0.000 | 0.000 | 0 |
|  | Dicten20 | 4 | 0.520 | 0.416 | -0.282 |
|  | Dicten25 | 2 | 0.120 | 0.113 | -0.062 |
|  | Dicten27* | 4 | 0.417 | 0.568 | 0.166 |
|  | Dicten29 | 4 | 0.320 | 0.282 | -0.167 |
| **11** | Dicten02 | 5 | 0.278 | 0.253 | -0.142 |
| n = 18 | Dicten11* | 2 | 0.500 | 0.375 | -0.293 |
|  | Dicten18 | 3 | 0.167 | 0.156 | -0.085 |
|  | Dicten20 | 2 | 0.056 | 0.054 | -0.028 |
|  | Dicten25 | 2 | 0.222 | 0.198 | -0.118 |
|  | Dicten27* | 4 | 0.235 | 0.517 | 0.276* |
|  | Dicten29 | 3 | 0.222 | 0.204 | -0.114 |
| **12** | Dicten02 | 3 | 0.500 | 0.383 | -0.286 |
| n = 28 | Dicten11* | 4 | 0.714 | 0.562 | -0.160 |
|  | Dicten18 | 2 | 0.036 | 0.035 | -0.018 |
|  | Dicten20 | 3 | 0.143 | 0.135 | -0.073 |
|  | Dicten25 | 2 | 0.036 | 0.035 | -0.018 |
|  | Dicten27* | 4 | 0.429 | 0.714 | 0.200* |
|  | Dicten29* | 1 | 0.000 | 0.000 | 0 |
